# Weighted Single-Step Genome-Wide Association Study Identifies Candidate Genes for Carcass Traits and Primal Cut Yields in Hanwoo Cattle

**DOI:** 10.3390/ani16010136

**Published:** 2026-01-03

**Authors:** Jun Park, Ji Suk Yu, Sun Kyu Byun, Ho Sung Choe, Do Hyun Kim

**Affiliations:** 1 Dasan Pig Breeding Co., Namwon 55716, Republic of Korea; jpbreed90@gmail.com; 2 Department of Animal Biotechnology, Jeonbuk National University, Jeonju 54896, Republic of Korea; kusizu0529@gmail.com; 3 Animal Genetic Resources Research Center, National Institute of Animal Science, Rural Development Administration, 224, Hamyang 50000, Republic of Korea; bsk0311@korea.kr

**Keywords:** Hanwoo cattle, carcass traits, primal cut yields, weighted single-step genome-wide association study, genomic selection

## Abstract

This study identified genomic regions associated with carcass traits and primal cut yields in Hanwoo cattle using weighted single-step genome-wide association study (WssGWAS). A total of 50,227 carcass records and genomic data from 23,573 animals with 45,057 single-nucleotide polymorphisms were analyzed. Heritability estimates were 0.24 for carcass weight, 0.22 for eye muscle area, 0.31 for backfat thickness, and 0.36 for marbling score, while those for primal cut yields ranged from 0.02 to 0.26. For carcass traits, candidate genes were identified for carcass weight (*XKR4* 2.35%, *COBL* 1.26%), eye muscle area (*LCORL* 1.56%, *TGFBR2* 1.49%), backfat thickness (*ATG7* 1.27%, *MYPN* 1.33%), and marbling score (*TWIST2* 1.16%, *BMP4* 1.14%). For primal cut yields, the chromosome 6 region containing WDR1 was commonly identified across six traits and the chromosome 4 region containing *CACNA2D1* across five traits; the chromosome 28 region containing *SIRT1* explained the highest genetic variance (6.46%) for striploin. These pleiotropic regions are potential targets for genomic selection to improve production efficiency and carcass value in Hanwoo.

## Simple Summary

The economic value of Hanwoo carcasses is determined not only by marbling score but also by primal cut yields. However, the genetic basis of primal cut yields remains largely unknown. This study identified genomic regions associated with carcass traits and primal cut yields. For carcass traits, candidate genes were identified for carcass weight (*XKR4*, *COBL*), eye muscle area (*LCORL*, *TGFBR2*), backfat thickness (*ATG7*, *MYPN*), and marbling score (*TWIST2*, *BMP4*). For primal cut yields, the chromosome 6 region containing WDR1 was commonly identified across six traits and the chromosome 4 region containing CACNA2D1 across five traits, suggesting pleiotropic effects. These findings provide candidate genomic regions that may inform future breeding strategies aimed at improving both carcass quality and meat yield, pending validation in independent populations.

## 1. Introduction

Recent developments in genomic technologies have enabled livestock breeders to incorporate dense marker information into evaluation systems alongside traditional pedigree-based approaches. In South Korea, government-supported programs have accelerated this transition by establishing large reference populations and encouraging the use of genomic selection (GS) in major livestock species. As genotyping costs continue to decrease, genomic prediction is gradually replacing conventional best linear unbiased prediction (BLUP) evaluations, improving the accuracy of estimated breeding values (EBVs) and contributing to faster genetic gain [1]. Continued refinement of GS methodologies, validation procedures, and single-nucleotide polymorphism (SNP) panel design has further strengthened the reliability of genomic evaluations [2].

Hanwoo, the native beef breed of Korea, is known for its high marbling, tenderness, and characteristic flavor [3]. Korean consumers place substantial value on premium grilling cuts such as tenderloin, sirloin, and striploin, which are priced higher than cuts like chuck or brisket [4,5]. Therefore, economic returns are influenced not only by carcass weight or marbling but also by the yield and composition of individual primal cuts. Genome-wide association studies (GWAS) have been widely used to identify genomic regions associated with economically important traits in livestock [6]. However, single-marker GWAS models may not fully characterize the polygenic architecture of quantitative traits. Methods that evaluate all markers simultaneously, such as weighted single-step GWAS (WssGWAS), can better account for linkage disequilibrium and distributed genetic effects while integrating phenotypic information from non-genotyped relatives [7,8].

In Hanwoo populations, phenotypic and genomic data have been collected from different groups of animals. Carcass measurements have accumulated over many years from slaughtered cattle, whereas genotyping has been carried out mainly on breeding females. Recent provincial genotyping programs have further expanded female reference populations, resulting in limited overlap between animals with carcass phenotypes and those with genomic records. This structure may reduce the accuracy of variance component estimates and limit the power of GWAS methods based on deregressed EBVs or pseudo-phenotypes [9].

This data structure requires analytical approaches that can integrate pedigree, genotype, and phenotype information while maintaining statistical power in unbalanced datasets. The weighted single-step GWAS (WssGWAS) method is well suited to this context. As an extension of the single-step genomic BLUP framework, it allows the identification of associated genomic regions in populations with unbalanced phenotypic and genotypic data [7,8]. This feature is particularly relevant in Hanwoo, where carcass phenotypes and genomic data originate from different subsets of the population.

The objective of this study was to identify genomic regions and candidate genes associated with carcass traits and primal cut yields in Hanwoo cattle using the WssGWAS approach. By combining extensive carcass records with genomic and pedigree information—much of it collected from breeding females—we aimed to describe the genetic architecture underlying carcass composition and the yield of economically important primal cuts. The results are expected to support genomic evaluation strategies suited to the current structure of Hanwoo breeding programs and contribute to improvements in meat quality and genetic progress.

## 2. Materials and Methods

### 2.1. Animal and Phenotype Data

A total of 50,227 carcass records were included in this study. Carcass and primal cut data were obtained from official slaughter records accessed through the Open Application Programming Interface (OpenAPI) service of the Korea public data portal (https://www.data.go.kr; accessed on 30 December 2025). All records were evaluated according to the standardized grading protocols of the Korea Institute for Animal Products Quality Evaluation (KAPE). These protocols define uniform carcass grading criteria, primal cut cutting procedures, measurement guidelines, and equipment calibration, and all evaluations are conducted by trained and certified personnel across certified slaughter facilities. Records classified as “unqualified” in the official carcass grading system were excluded prior to analysis.

The initial dataset comprised 23,942 genotyped Hanwoo cattle participating in a regional genetic improvement program in Jeonbuk Province, South Korea, conducted between 2022 and 2024. Phenotypic records were expanded to include related animals, covering up to three generations of ancestors and descendants. This procedure resulted in a final dataset of 50,227 animals with available carcass records.

Pedigree information was obtained from the Korea Animal Improvement Association (KAIA). The complete pedigree file contained 79,077 animals, including 14,685 founders, with a pedigree depth extending up to 12 generations.

The carcass traits analyzed were carcass weight (CW), eye muscle area (EMA), backfat thickness (BF), and marbling score (MS). Eleven primal cut yield traits were also evaluated: tenderloin (TLN), sirloin (SLN), striploin (STLN), chuckroll (CHK), shoulder (SLD), top round (TRD), bottom round (BRD), brisket (BSK), shank (SK), rib (RB), and total primal cut yield (TPC). Primal cut yields were expressed as percentages of carcass weight.

### 2.2. Genotype Data

Animals were genotyped using the Illumina Bovine SNP50K BeadChip (Bovine SNP 50K) customized for Hanwoo cattle. The initial genotyping dataset consisted of 53,866 SNPs from 23,942 animals. Quality control was performed using PLINK software (v1.90) [10]. Animals with call rates below 0.90 were excluded, resulting in the removal of 368 individuals. SNPs were filtered based on call rate (<0.90; 696 SNPs removed), minor allele frequency (<0.01; 4831 SNPs removed), Hardy–Weinberg equilibrium (*p* < 10^−7^; 1503 SNPs removed), and non-autosomal loci (1671 SNPs removed). After quality control, 45,057 autosomal SNPs from 23,573 animals were retained for subsequent analyses.

Among the 50,227 animals with carcass records, 16,332 animals (32.5%) were genotyped.

### 2.3. Statistical Analysis

Variance components for carcass traits and primal cut yields were estimated using the average information restricted maximum-likelihood (AIREML) method under a single-trait animal model. The single-step genomic best linear unbiased prediction (ssGBLUP) approach was used to estimate genomic estimated breeding values (GEBVs), following the model:

(1) y=Xb+Za+e

where y is the vector of phenotypic observations, X is the incidence matrix for fixed effects, and b is the vector of fixed effects (herd-birth year-birth season, sex); slaughter age was included as a covariate for each trait. Z is the incidence matrix for random additive effects, a is the vector of additive genetic effects, and e is the vector of residuals. Heritability was estimated as $h^{2}=\;\frac{\sigma_{a}^{2}}{\sigma_{a}^{2}+\sigma_{e}^{2}}$, where $\sigma_{a}^{2}$ and $\sigma_{e}^{2}$ represent additive genetic and residual variances, respectively.

The weighted single-step GWAS (WssGWAS) method was used to identify genomic regions and candidate genes associated with carcass and primal cut yield traits. Unlike conventional BLUP models, ssGBLUP replaces the inverse of the pedigree-based relationship matrix ($A^{-1}$) with a combined pedigree–genomic matrix ($H^{-1}$), defined as follows [11]:

(2) $$H^{-1}=A^{-1}+\left[\begin{array}{cc} 0 & 0\\ 0 & G^{-1}-A_{22}^{-1} \end{array}\right]$$

where A is the numerator relationship matrix for all animals; $A_{22}$ is the pedigree-based relationship matrix for genotyped animals, and G is the genomic relationship matrix [12], calculated as:

(3) $$G=\frac{{ZDZ}^{\prime }}{\sum_{i=1}^{M}2p_{i}(1-p_{i})}$$

where Z is the matrix of gene content adjusted for allele frequency (0, 1, or 2 for *aa*, *Aa*, and *AA* genotypes), D is a diagonal matrix of SNP-specific weights (initially D=I), M is the total number of SNPs, and $p_{i}$ is the minor allele frequency of the $i^{th}$ SNP. Estimates of SNP effects and weights for WssGWAS were obtained according to following steps [7]:

1. In the first iteration (t = 1): $D=I;\;G_{\left(t\right)}=D_{\left(t\right)}Z^{\prime }\lambda$, where $\lambda =\frac{1}{\sum_{i=1}^{M}2p_{i}(1-p_{i})}$ [7];
2. GEBVs were calculated for the entire dataset using ssGBLUP;
3. GEBVs were converted to estimates of SNP effects $\left(\hat{u}\right):\;\hat{u}=\lambda D_{\left(t\right)}Z^{\prime }G_{\left(t\right)}^{-1}{\hat{a}}_{g}$, where ${\hat{a}}_{g}$ is the GEBVs of animals that were also genotyped;
4. The weight for each SNP to be used in the next iteration was calculated as: $d_{i(t+1)}={\hat{u}}_{i\left(t\right)}^{2}2p_{i}(1-p_{i})$, where i is the $i^{th}$ SNP;
5. the SNP weights were normalized to keep the total genetic variance constant:
  (4) $$D_{\left(t+1\right)}=\frac{tr(D_{\left(1\right)})}{tr(D_{\left(t+1\right)})}D_{\left(t+1\right)}$$
6. $G_{\left(t+1\right)}=ZD_{\left(t+1\right)}Z^{\prime }\lambda$ was calculated;
7. t=t+1 and loop to step 2.

The procedure was iterated three times, following common practice in WssGWAS studies and considering the exploratory objective of identifying candidate genomic regions [13,14]. At each iteration, weights for SNPs were updated (steps 4 and 5), and used to construct the G matrices (step 6), update GEBVs (step 2), and estimate SNP effects (step 3). The percentage of genetic variance explained by the $i^{th}$ each set of consecutive SNPs ($i^{th}$ SNP window) was calculated [14]. In this study, SNPs located within 1.0 Mb [15], and the percentage of genetic variance explained by each 1.0 Mb window was calculated as follows:

(5) $$\frac{Var(a_{i})}{\sigma_{a}^{2}}\times 100=\frac{Var(\sum_{j=1}^{x}Z_{j}{\hat{u}}_{j})}{\sigma_{a}^{2}}$$

where $a_{i}$ is the genetic value of the $i^{th}$ SNP window that consists of a region of consecutive SNPs located within 1.0 Mb; $\sigma_{a}^{2}$ is the total additive genetic variance; $Z_{j}$ is the vector of gene content of the $j^{th}$ SNP for all individuals and ${\hat{u}}_{j}$ is the effect of the $j^{th}$ SNP within the $i^{th}$ window. To visualize the distribution of these SNP windows, Manhattan plots were generated using the R v4.4.3 [16] and CMplot package [17]. The procedures mentioned above were run with BLUPF90 software family programs [18] iteratively. The RENUMF90 (v1.168) module was used to obtain the required parameter file formats; the BLUPF90+ (v2.63) module was used for variance components estimation and GEBVs calculation, and the postGSF90 (v1.86) module for association analysis.

We aimed to identify specific genomic regions associated with carcass traits and primal cut yield by examining quantitative trait loci (QTL) using genomic windows that accounted for more than 1.0% of the total genetic variance. These genomic windows, previously employed in similar studies [19], represent regions of the genome that contribute significantly to the genetic variation underlying carcass traits and primal cut yield. Since the 1.0 Mb window explained on average 0.0511% (100% divided by 1956 genomic regions) of the genetic variance, the 1% threshold is over 19.5 times the expected average genetic variance explained by the 1.0 Mb window [2]. For the identified QTL regions, genes were searched using the Ensembl ARS-UCD 1.3 (https://asia.ensembl.org/Bos_taurus/Info/Index?; accessed on 30 December 2025) database within significant windows. Cattle QTL information was retrieved from the Animal QTLdb (https://www.animalgenome.org/cgi-bin/QTLdb/BT/index; accessed on 30 December 2025) to compare with the significant regions identified in this study. SNP-level genetic variance within significant windows is reported in Supplementary Tables S1–S15.

## 3. Results and Discussion

### 3.1. Descriptive Statistics and Heritability for the Carcass Traits and Primal Cut Yields

The reference population used in this study consisted mainly of breeding cows, leading to an older average slaughter age (63.9 months) than that observed in typical Hanwoo steer populations (Table 1). This reflects recent large-scale genotyping efforts that have focused on Hanwoo cows since 2022 and should be considered when interpreting the results. Because cows generally produce lighter carcasses and lower marbling scores than steers, the mean carcass performance in this dataset was lower than values reported in previous studies based on commercial steers [20].

The distribution of primal cut yields followed patterns expected for Hanwoo carcasses, with ribs, brisket, and sirloin contributing the largest proportions and tenderloin the smallest. Although tenderloin accounts for a relatively small share of the carcass, it retains the highest market value, followed by striploin and sirloin. These price differences reflect consumer preference for premium grilling cuts and emphasize the economic relevance of improving the yield of these portions through genetic selection.

Heritability estimates indicated moderate genetic control for BF and MS, whereas CW and EMA showed lower estimates. These results are consistent with the expectation that traits related to fat deposition respond more predictably to genomic selection. Heritability estimates for primal cut yields ranged from low to moderate (0.02–0.26), suggesting that both genetic and environmental factors contribute substantially to their variation. TPC showed a heritability comparable to major carcass traits, indicating that it may serve as a useful indicator in future breeding programs (Table 1).

Although genetic correlations were not estimated in this study, previous reports have described strong genetic associations between carcass fat traits and primal cut yields [21,22]. These findings imply that indirect selection for primal cut yields could be achieved through improvement of carcass traits with higher heritability, offering a practical strategy for enhancing Hanwoo under current breeding conditions.

### 3.2. WssGWAS of Carcass Traits

#### 3.2.1. Carcass Weight

The WssGWAS analysis for carcass weight identified significant genetic variance contributions on several chromosomes. The strongest association signal was identified in the 22.53–23.50 Mb region on *Bos taurus* autosome (BTA) 14, where *RGS20* and *XKR4* explained 2.35% of the total genetic variance (Table 2). The adjacent region at 24.58–25.33 Mb on BTA14 has been reported to explain 17.66% of CW genetic variance and 7.98% of EMA genetic variance in Hanwoo [23], consistent with our findings.

*XKR4* has been reported as a positional candidate gene associated with feed intake and growth traits in cattle [24], and showed a significant association with subcutaneous fat thickness in *Bos indicus* and crossbred cattle, explaining approximately 5.9% of genetic variance [25]. The 23–26 Mb region on BTA14 has been repeatedly identified as a pleiotropic QTL for body weight and carcass traits in more than 10 U.S. beef cattle breeds, including Angus, Hereford, Simmental, and Gelbvieh [26]. This region is a conserved locus affecting growth traits across *Bos taurus* breeds.

In the 4.65–5.64 Mb region on BTA4, the *COBL* gene was identified. *COBL* has been associated with a missense SNP (rs210475204) for CW in Hanwoo, explaining 1.634% of CW genetic variance [27]. *COBL* functions as an actin nucleator through its WASP-homology 2 (WH2) domain, participating in axis formation during development and regulating cytoskeletal dynamics via barbed-end dynamics [28,29]. The actin cytoskeleton is essential for myofiber formation and tissue growth, so *COBL* function is biologically relevant to carcass weight variation.

The 35.38–36.37 Mb region on BTA6 contained *FAM13A*, *HERC3*, and *GPRIN3*, while the 38.06–39.03 Mb region on BTA6 contained *SLIT2*. The 37–42 Mb region on BTA6 contains the *NCAPG* and *LCORL* genes and has been reported as a major QTL region associated with feed intake, growth rate, carcass weight, and eye muscle area in several cattle breeds [30,31]. The BTA6 regions identified in this study are adjacent to this QTL region, and this chromosome likely contributes to growth traits in Hanwoo.

In summary, CW in Hanwoo is influenced by genetic loci for skeletal development, metabolic regulation, and tissue growth. The concentration of strong signals on BTA14 and BTA6 is consistent with previous multi-breed studies indicating that these chromosomes contain major QTLs for growth traits in cattle [26,32]. These findings support genomic selection strategies to improve carcass weight in Hanwoo breeding programs.

#### 3.2.2. Eye Muscle Area

Significant regions for EMA were identified on BTA6, 17, 22, and 25. The 37.91–38.88 Mb region on BTA6, containing *LCORL* and *DCAF16*, explained the highest genetic variance (1.56%) (Table 2). This region was identified as a common locus explaining height variation in a meta-analysis of 58,265 cattle [33] and represents a major QTL region significantly associated with eye muscle area and lean growth in crossbred beef cattle [30]. Transcript levels of *DCAF16* and *NCAPG* are significantly associated with average daily gain (ADG), and these genes influence growth traits through differential expression in muscle tissue [34].

*SMAD1* was identified in the 12.73–13.71 Mb region on BTA17 (1.32%) and regulates the bone morphogenetic protein (BMP) signaling pathway. The BMP pathway promotes skeletal muscle hypertrophy by mediating mechanistic target of rapamycin (mTOR) signaling activation through Smad1/5 [35]. *TGFBR2* (1.49%), identified in the 5.17–6.15 Mb region on BTA22, functions as a transforming growth factor-beta (TGF-β) receptor that mediates TGF-β signaling to inhibit myoblast fusion and regulate myofiber size [36].

The 38.01–39.00 Mb region on BTA25 contained *ACTB* (1.21%). *ACTB* is a major structural protein of the cytoskeleton and is essential for maintaining cell shape and motility [37]. Actin filaments provide mechanical support and locomotor force to cells, so variation in *ACTB* may affect muscle tissue structure.

In summary, EMA is influenced by genetic loci for skeletal muscle development, cell differentiation, and tissue structure. The *LCORL*-*DCAF16* region on BTA6 has been established as a common genetic determinant of body size and growth across mammals [33], while BMP and TGF-β signaling genes (*SMAD1*, *TGFBR2*) regulate muscle differentiation and fusion [35,36]. *LCORL*, *DCAF16*, *SMAD1*, *TGFBR2*, and *ACTB* are potential targets for genomic selection to improve eye muscle area in Hanwoo breeding.

#### 3.2.3. Backfat Thickness

Significant genomic regions for BF were identified on BTA22 and BTA28. *ATG7* and *VGLL4* were identified in the 55.71–56.64 Mb region on BTA22 (1.27%), while *MYPN*, *TET1*, *SIRT1*, and *HERC4* were identified in the 24.93–25.88 Mb region on BTA28 (1.33%) (Table 2).

*ATG7* regulates autophagy and is involved in cellular homeostasis and protein metabolism. During the fattening period in Japanese Black cattle, *ATG7* expression increased along with skeletal muscle growth, and its elevated expression in the longissimus muscle showed strong correlations with ultrasound eye muscle area and body weight [38]. Autophagy is therefore important for muscle growth and body composition.

*MYPN*, identified on BTA28, maintains sarcomere structure and regulates Z-line organization. In Chinese beef cattle populations, the A1795G SNP in the *MYPN* gene showed significant associations with ultrasound eye muscle area and carcass eye muscle area [39].

*TET1* functions as a DNA demethylase and influences muscle differentiation through epigenetic regulation. *Myostatin* (*MSTN*) mutations reduce *SMAD2/SMAD3* activity, thereby releasing repression of the TET1 promoter and increasing *TET1* expression. This leads to demethylation of myogenic genes including *PAX3*, *PAX7*, *MyoD*, and *MyoG*, promoting muscle differentiation [40].

BF is determined by the balance between energy metabolism, fat deposition, and muscle development. *ATG7*, *MYPN*, and *TET1* influence body composition via autophagy, muscle structure maintenance, and epigenetic regulation. These genes are candidates for genomic selection to improve BF in Hanwoo breeding.

#### 3.2.4. Marbling Score

WssGWAS analysis for MS identified four significant genomic regions on BTA3, BTA5, BTA10, and BTA22 (Table 2). Among these, BTA22 (31.48–32.46 Mb) exhibited the highest genetic variance at 1.24%, with *MDFIC2* and *MITF* identified as positional candidates in this region.

*TWIST2* and *BMP4* are associated with adipogenesis regulation. *TWIST2*, located on BTA3 (117.33–118.30 Mb), is a basic helix–loop–helix (bHLH) family transcription factor that inhibits adipogenesis in 3T3-L1 cells and primary preadipocytes [41]. *TWIST2* knockdown increases lipid accumulation while overexpression reduces it [41], confirming its direct role in fat deposition. *BMP4* on BTA10 (66.33–67.31 Mb) is a morphogen that induces adipocyte lineage commitment of mesenchymal stem cells through *SMAD1/5/8* signaling [42,43]. In cattle, *BMP4* promotes white adipogenesis [44], supporting its association with MS.

*LRRFIP1* on BTA3 is a notable candidate related to muscle development. In Nelore cattle, *LRRFIP1* was reported as a hub transcript associated with eye muscle area and regulates smooth muscle cell proliferation and actin filaments [45]. Myogenesis and adipogenesis originate from common progenitor cells and compete with each other [44], so *LRRFIP1* may affect intramuscular fat deposition.

The functional associations of *TBC1D22A* on BTA5 and *MITF* on BTA22 with adipogenesis or meat quality have not been characterized. *MITF* regulates melanocyte development, but no studies have investigated its role in intramuscular fat in cattle. These genes are novel candidates and require further functional validation.

**Figure 1:**
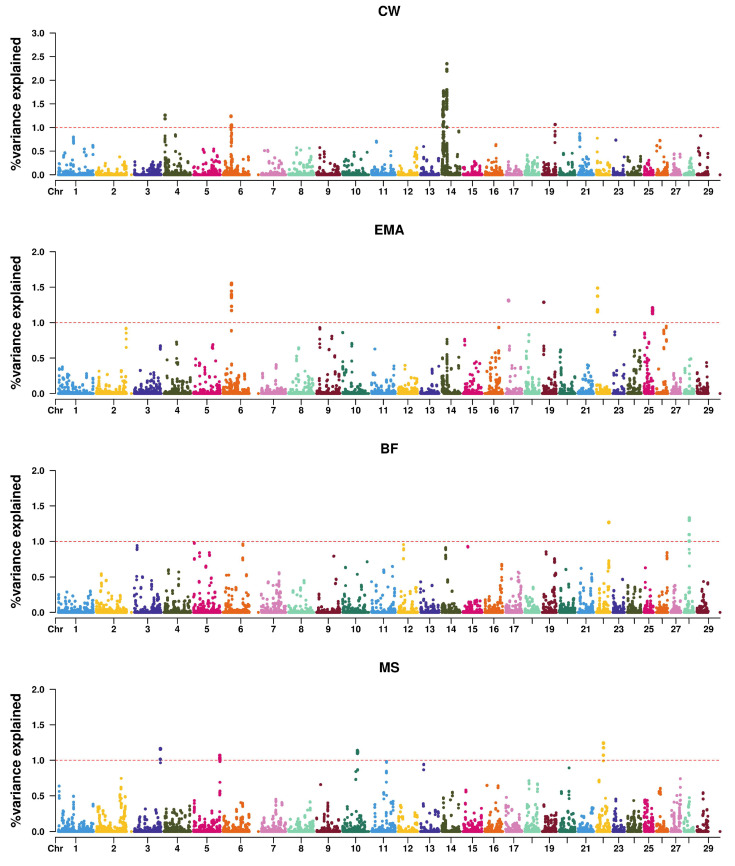
Manhattan plots showing the proportion of genetic variance explained by 1.0 Mb windows for carcass traits. CW, carcass weight; EMA, eye muscle area; BF, backfat thickness; MS, marbling score. The red dashed line indicates the significance threshold of 1% genetic variance. gVar (%) represents the percentage of additive genetic variance explained by each genomic window.

The MS-associated regions in this study do not overlap with BTA2, BTA12, BTA16, and BTA24 previously reported in Hanwoo GWAS [46]. This discrepancy is likely due to differences in reference population composition. Our study analyzed a population primarily composed of breeding cows rather than commercial steers, which may have limited the ability to detect QTLs associated with MS. However, identifying adipogenesis-related genes (*TWIST2*, *BMP4*) and muscle development-related genes (*LRRFIP1*) improves understanding of the genetic architecture of MS in Hanwoo. Manhattan plots for carcass traits are presented in Figure 1.

### 3.3. WssGWAS of Primal Cut Yields

Primal cut yield traits showed significant genomic signals across several chromosomes, with multiple 1 Mb windows exceeding the 1% genetic variance threshold. Unlike major carcass traits that have been extensively investigated across cattle breeds, primal cut yields have received limited attention in genomic studies, largely due to the recent availability and inherent complexity of large-scale phenotyping for individual cuts. Consequently, many candidate genes identified in this study were interpreted based on their known biological functions and mechanistic relevance to tissue composition, rather than on direct phenotypic associations reported in previous studies. Accordingly, candidate gene interpretations for primal cut yield traits should be regarded as functional hypotheses rather than direct evidence of causality. Manhattan plots for primal cut yields are presented in Figure S1.

In this study, pleiotropy refers to overlapping genomic regions associated with multiple traits and should be interpreted as shared genetic signals rather than formally tested pleiotropic effects.

#### 3.3.1. Premium Cuts

The premium cuts—tenderloin, sirloin, and striploin—command the highest market prices among Hanwoo cuts. This study identified several genomic windows explaining over 1% of genetic variance for these three traits, with the 105.60–106.57 Mb region on BTA6 commonly identified across all three.

This BTA6 region contains *WDR1*, which cooperates with cofilin to promote actin filament disassembly and is involved in structural remodeling of muscle cells. *CFL2* (cofilin-2) showed significant associations with body mass, chest girth, and hip length in Jinchuan cattle [47], and the *WDR1*-cofilin pathway likely contributes to premium cut yield through muscle development.

The 23.62–24.60 Mb region on BTA28, which exhibited the highest genetic variance for striploin (6.46%), contains *SIRT1* and *MYPN*. *SIRT1* has been reported to inhibit adipogenic differentiation of intramuscular preadipocytes through reduced H3K4 acetylation [44]. *MYPN* maintains sarcomere structure, regulates Z-line organization, and shows significant associations with eye muscle area and water-holding capacity in cattle [39]. The BTA28 region may therefore influence striploin yield by balancing muscle structure and fat deposition.

*GSK3B*, located in the 65.06–66.06 Mb region on BTA2 (STLN, 2.12%), regulates the Wnt/β-catenin signaling pathway, inhibiting adipogenic differentiation and promoting myotube formation in fibro/adipogenic progenitors (FAPs) of bovine skeletal muscle [48]. *GSK3B* may therefore contribute to striploin yield variation by regulating muscle-to-fat ratio.

#### 3.3.2. Round Cuts

Top round and bottom round, as hindquarter cuts with substantial contribution to meat yield, shared identical genomic windows. Both traits showed significant genetic variance in the 105.60–106.57 Mb region on BTA6 (TRD 1.50%, BRD 1.66%) and the 55.55–56.48 Mb region on BTA17 (TRD 1.21%, BRD 1.28%).

The 105.60–106.57 Mb region on BTA6 contains *WDR1* and *SLC2A9*. *WDR1* cooperates with cofilin to regulate actin dynamics [47]. This region was identified in both premium and hindquarter cuts, indicating that muscle structural remodeling contributes to yield across different anatomical locations.

The 55.55–56.48 Mb region on BTA17 contains genes for energy metabolism and muscle homeostasis, including *PRKAB1*, *ACAD10*, and *HSPB8*. *PRKAB1* encodes the β1 regulatory subunit of AMP-activated protein kinase (AMPK), and the AMPK pathway has been reported to regulate intramuscular fat accumulation and glycogen levels in bovine muscle [49]. *ACAD10* belongs to the acyl-CoA dehydrogenase family and is involved in mitochondrial fatty acid β-oxidation. *HSPB8*, a member of the small heat shock protein family, has been reported to protect myofibers from postmortem proteolysis in bovine skeletal muscle [50]. The energy metabolism and muscle protection functions of these genes may influence muscle mass and composition in hindquarter cuts.

The two hindquarter cuts share identical QTL regions, likely due to their anatomical proximity and similar muscle characteristics. This pleiotropic effect can improve selection efficiency for meat yield.

#### 3.3.3. Forequarter Cuts

Chuckroll, shoulder, and brisket are forequarter cuts that share characteristics of high connective tissue content and influence from skeletal structure. These three traits exhibited several common QTL regions, with the same regions on BTA4 and BTA19 repeatedly identified.

The 38.07–39.06 Mb region on BTA4 commonly explained over 1% of genetic variance for five traits: SLN, CHK, SLD, BSK, and TPC. *CACNA2D1*, located in this region, encodes the α2/δ subunit of voltage-gated calcium channels and shows significant associations with carcass weight, dressing percentage, and backfat thickness in cattle [51]. Calcium signaling is central to muscle contraction and intracellular signaling, so the pleiotropic effects of *CACNA2D1* may contribute to muscle development in various cuts.

The 7.33–8.33 Mb region on BTA19 was identified in SLD (1.14%) and BSK (1.32%), containing *NOG* and *ANKFN1*. *NOG* functions as a BMP antagonist that inhibits chondrocyte proliferation and regulates joint formation [52]. Forequarter cuts are closely associated with shoulder and thoracic skeletal structures, so *NOG* may influence yield proportions of these cuts by regulating skeletal development.

The 8.22–9.22 Mb region on BTA28 was identified in several traits including chuck, shoulder, brisket, and rib, containing extracellular matrix and tissue development genes such as *LYST*, *NID1*, and *EDARADD*. These common QTLs were repeatedly identified, indicating that forequarter cuts share similar genetic foundations due to anatomical proximity and similar connective tissue characteristics.

#### 3.3.4. Shank and Rib

Shank and rib are cuts with high connective tissue content, typically used for slow cooking. Both QTL regions identified for SK (105.60–106.57 Mb on BTA6 and 55.55–56.48 Mb on BTA17) were shared with other primal cuts, indicating that genes in these regions broadly influence primal cut yield.

RB showed the most QTL regions among all traits examined, with over ten 1 Mb windows explaining more than 1% of genetic variance. *COL1A1*, located at the 36.99–37.95 Mb region on BTA28 (1.63%), encodes the α1 chain of Type I collagen and is a major component of intramuscular connective tissue. Collagen content and cross-linking directly affect meat tenderness in cattle [53]. As rib is a connective-tissue-rich cut, differences in *COL1A1* expression may contribute to the composition and yield of this cut.

*PINK1* was identified at the 131.94–132.93 Mb region on BTA2 (1.45%). *PINK1* is a kinase that regulates mitochondrial quality control and mitophagy, and recent porcine studies have reported negative correlations between *PINK1* expression and intramuscular fat content, with knockdown increasing intramuscular fat (IMF) accumulation [54]. This mitochondrial regulatory function may influence the fat-to-muscle composition of the rib cut.

Several QTLs were shared between RB and other cuts. *NOG* at the 7.33–8.33 Mb region on BTA19 was commonly identified in SLD, BSK, and RB, indicating broad involvement in skeletal-related cut yield, while the 8.34–9.22 Mb region on BTA28 was shared among CHK, SLD, BSK, and RB. These shared QTL patterns indicate that anatomically adjacent cuts share common genetic foundations.

#### 3.3.5. Total Primal Cut Yield

Total primal cut yield is the overall proportion of major primal cuts from the carcass and a key indicator of dressing percentage. Two QTL regions explaining over 1% of genetic variance were identified for TPC, both shared with individual primal cuts.

The 38.07–39.06 Mb region on BTA4 (1.12%) was also identified in SLN, CHK, SLD, and BSK, containing *CACNA2D1*. As described earlier, *CACNA2D1* showed significant associations with carcass weight, dressing percentage, and backfat thickness in cattle including Hanwoo [51], and this gene likely contributes to overall primal cut yield.

The 105.60–106.57 Mb region on BTA6 (1.02%) was the most frequently identified region in this study, explaining over 1% of genetic variance for six traits including TLN, SLN, TRD, BRD, and SK. This region contains *WDR1*, *SLC2A9*, and *CLNK*, and *WDR1* regulates actin dynamics in cooperation with cofilin [47]. This region was commonly identified across multiple primal cuts and likely influences overall muscle development and primal cut composition.

Both QTLs identified for TPC were shared with individual primal cuts, indicating that total primal cut yield reflects the summative characteristics of individual cut yields. Genetic improvement of TPC can therefore be efficiently achieved by selecting for genes in shared QTL regions.

Although strong genetic correlations are expected among carcass traits and primal cut yields, the present study employed single-trait WssGWAS models to accommodate the computational demands of analyzing 15 traits in a large population. This approach was chosen to ensure stable estimation under the unbalanced structure of phenotypic and genotypic data in the Hanwoo population.

## 4. Conclusions

This study identified genomic regions associated with carcass traits and 11 primal cut yield traits in Hanwoo cattle using WssGWAS. For carcass traits, previously reported QTL regions were confirmed, including the BTA14 region containing *XKR4* for carcass weight and the BTA6 region containing *LCORL* for eye muscle area. For primal cut yields, the genomic region from 105.60 to 106.57 Mb on BTA6 containing *WDR1* was identified across six traits (tenderloin, sirloin, top round, bottom round, shank, and total primal cut yield), and the region from 38.07 to 39.06 Mb on BTA4 containing *CACNA2D1* was detected across five traits (sirloin, chuckroll, shoulder, brisket, and total primal cut yield). These pleiotropic regions represent potential targets for genomic selection to simultaneously improve yields of multiple primal cuts.

As the first large-scale GWAS for primal cut yields in Hanwoo cattle, this study provides foundational genomic information for developing balanced selection indices that consider both meat quality and carcass yield. Validation in independent populations and functional characterization of candidate genes are warranted to confirm these findings.

## Figures and Tables

**Table 1:** Description, variance components, and genetic parameters of carcass traits and primal cut yields in Hanwoo cattle.

Traits ^1^	Mean	SD ^2^	Min	Max	CV (%) ^3^	$\sigma_{a}^{2}$ *	$\sigma_{e}^{2}$ *	$h^{2}$ (SE) *
**Slaughter Age (month)**	63.9	32.04	11.20	283.63	50.09	**-**	**-**	**-**
**Carcass Trait**								
CW (kg)	378.39	76.51	107	744	20.22	669.04	2128.1	0.24 (0.02)
EMA (cm^2^)	87.64	14.94	20	197	17.05	32.47	113.04	0.22 (0.02)
BF (mm)	12.83	5.90	1	88	45.99	8.95	20.31	0.31 (0.02)
MS (score)	4.57	2.2	1	9	49.02	1.19	2.15	0.36 (0.02)
**Primal cut yield**								
TLN (%)	1.80	0.06	1.69	1.83	3.33	0.0004	0.0023	0.16 (0.02)
SLN (%)	10.13	0.50	9.31	10.74	4.94	0.0589	0.1675	0.26 (0.02)
STLN (%)	2.35	0.05	2.28	2.39	2.13	0.0001	0.0019	0.02 (0.00)
CHK (%)	4.04	0.25	3.67	4.39	6.19	0.0151	0.0454	0.25 (0.02)
SLD (%)	7.10	0.33	6.67	7.57	4.65	0.0239	0.0769	0.24 (0.02)
TRD (%)	6.00	0.23	5.58	6.22	3.83	0.0117	0.0351	0.25 (0.02)
BRD (%)	9.51	0.32	8.91	9.78	3.36	0.0215	0.0683	0.24 (0.02)
BSK (%)	10.53	0.28	10.2	10.95	2.66	0.0168	0.0587	0.22 (0.02)
SK (%)	4.39	0.13	4.13	4.49	2.96	0.0037	0.0127	0.22 (0.02)
RB (%)	13.90	0.07	13.85	14.01	0.5	0.0006	0.0041	0.13 (0.01)
TPC (%)	69.74	2.07	66.32	72.29	2.97	1.0272	2.9209	0.26 (0.02)

Note: The number of animals was 50,227 for slaughter age, carcass traits, and primal cut yield traits. Among these, 16,332 animals were genotyped and used for genetic analyses. ^1^ carcass weight (CW), eye muscle area (EMA), backfat thickness (BF), marbling score (MS), tenderloin (TLN), sirloin (SLN), striploin (STLN), chuckroll (CHK), shoulder (SLD), top round (TRD), bottom round (BRD), brisket (BSK), shank (SK), rib (RB), and total primal cut yield (TPC). ^2^ Standard deviation. ^3^ Coefficient of variation. * Additive genetic variance, residual variance, and heritability (standard error).

**Table 2:** Genomic regions exceeding 1% of genetic variance and candidate genes for carcass traits and primal cut yields in Hanwoo cattle.

**Traits ^1^**	**BTA ^2^**	**Position (Mb)**	**gVar (%) ^3^**	**nSNP**	**Candidate Genes**
CW (kg)	4	4.65–5.64	1.26	31	*COBL*
	6	35.38–36.37	1.24	36	*FAM13A*, *HERC3*, *GPRIN3*, *ENSBTAG00000068460*, *ENSBTAG00000077377*, *ENSBTAG00000073697*
		38.06–39.03	1.05	32	*SLIT2*, *ENSBTAG00000076950*
	14	4.86–5.85	1.39	35	*COL22A1*, *FAM135B*, *KHDRBS3*
		6.11–7.11	1.38	29	*FAM135B*, *KHDRBS3*
		7.57–8.56	1.77	25	*KHDRBS3*, *bta-mir-30D*, *ZFAT*, *ST3GAL1*, *NDRG1*
		22.53–23.50	2.35	33	*RGS20*, *XKR4*
EMA (cm^2^)	6	37.91–38.88	1.56	35	*FAM184B*, *LCORL*, *DCAF16*, *SLIT2*, *ENSBTAG00000076950*, *ENSBTAG00000077372*
	17	12.73–13.71	1.32	14	*ZNF827*, *C4orf51*, *MMAA*, *SMAD1*, *ENSBTAG00000069004*, *OTUD4*, *ANAPC10*, *HHIP*
	22	5.17–6.15	1.49	29	*RBMS3*, *TGFBR2*, *ENSBTAG00000068006*
	25	38.01–39.00	1.21	21	*CYTH3*, *ZDHHC4*, *RNF216*, *ACTB*, *FBXL18*
BF (mm)	22	55.71–56.64	1.27	25	*ATG7*, *VGLL4*
	28	24.93–25.88	1.33	11	*DNAJC12*, *SIRT1*, *HERC4*, *MYPN*, *TET1*, *CCAR1*
MS (score)	3	117.33–118.30	1.16	21	*LRRFIP1*, *ERFE*, *ILKAP*, *TWIST2*, *ENSBTAG00000063693*
	5	117.93–118.93	1.07	24	*TBC1D22A*, *ENSBTAG00000056170*
	10	66.33–67.31	1.14	19	*BMP4*, *CGRRF1*, *SAMD4A*, *ENSBTAG00000078161*, *ENSBTAG00000078554*
	22	31.48–32.46	1.24	15	*MDFIC2*, *MITF*, *ENSBTAG00000075223*
TLN (%)	1	153.58–154.56	1.15	22	*PLCL2*, *TBC1D5*, *RFTN1*, *OXNAD1*
	2	23.17–24.15	1.37	22	*OLA1*, *CDCA7*
	6	105.60–106.57	2.49	22	*SLC2A9*, *WDR1*
		116.02–117.02	1.04	25	*ADD1*, *SH3BP2*, *TNIP2*
	17	55.55–56.48	1.20	21	*ACAD10*, *CCDC60*, *HSPB8*, *CIT*
	25	6.08–6.92	1.18	22	*RBFOX1*
	26	13.13–14.11	1.16	22	*ANKRD1*, *HECTD2*, *PCGF5*
SLN (%)	4	38.07–39.06	1.11	17	*PCLO*, *CACNA2D1*, *ENSBTAG00000070945*
	6	105.60–106.57	1.02	22	*CLNK*
STLN (%)	1	17.93–18.85	4.12	15	*NCAM2*, *TMPRSS15*
		65.06–66.06	2.12	18	*GSK3B*, *GPR156*, *FSTL1*, *STXBP5L*
	2	22.91–23.82	1.43	22	*OLA1*, *CDCA7*
	4	86.04–87.03	1.89	21	*PTPRZ1*, *AASS*, *CADPS2*
	6	98.34–99.27	1.18	22	*SCD5*, *SEC31A*, *COQ2*, *GPAT3*, *HELQ*
	10	55.84–56.84	3.18	19	*UNC13C*, *WDR72*
		82.19–83.17	6.46	20	*SLC39A9*, *PLEKHD1*, *SUSD6*, *SMOC1*, *SLC8A3*, *COX16*, *MED6*
	28	12.62–13.62	4.56	13	*BMS1*, *CHRM3*, *RET*, *CSGALNACT2*
		23.62–24.60	2.46	15	*CTNNA3*, *DNAJC12*, *SIRT1*, *HERC4*, *MYPN*
CHK (%)	4	20.19–21.15	1.24	29	*THSD7A*, *TMEM106B*
		38.07–39.06	1.28	17	*PCLO*, *CACNA2D1*, *ENSBTAG00000070945*
	26	47.61–48.57	1.01	17	*PTPRE*, *MKI67*, *MGMT*
	28	8.22–9.22	1.10	23	*TBCE*, *B3GALNT2*, *GNG4*, *LYST*, *NID1*, *GPR137B*, *ERO1B*, *EDARADD*
SLD (%)	4	20.18–21.15	1.48	29	*THSD7A*, *TMEM106B*, *ENSBTAG00000067048*
		38.07–39.06	1.28	17	*PCLO*, *CACNA2D1*, *ENSBTAG00000070945*
	19	7.33–8.33	1.14	19	*ANKFN1*, *NOG*
	26	47.61–48.57	1.00	17	*PTPRE*, *MKI67*, *MGMT*
	28	8.22–9.22	1.32	23	*TBCE*, *B3GALNT2*, *GNG4*, *LYST*, *NID1*, *GPR137B*, *ERO1B*, *EDARADD*
	29	30.17–31.15	1.09	20	*KIRREL3*, *ETS1*
TRD (%)	6	105.60–106.57	1.50	22	*SLC2A9*, *WDR1*
	17	55.55–56.48	1.21	21	*PRKAB1*, *ACAD10*, *CIT*, *HSPB8*
BRD (%)	6	105.60–106.57	1.66	22	*SLC2A9*, *WDR1*
	17	55.55–56.48	1.28	21	*PRKAB1*, *ACAD10*, *CIT*, *HSPB8*
BSK (%)	4	20.18–21.15	1.68	29	*THSD7A*, *TMEM106B*
		38.07–39.06	1.21	17	*PCLO*, *CACNA2D1*
	19	7.33–8.33	1.32	21	*ANKFN1*, *NOG*
	28	8.34–9.22	1.52	21	*TBCE*, *B3GALNT2*, *GNG4*, *LYST*, *NID1*, *GPR137B*, *ERO1B*, *EDARADD*
	29	30.17–31.15	1.26	20	*KIRREL3*, *ETS1*, *ENSBTAG00000076315*
SK (%)	6	105.60–106.57	1.84	22	*SLC2A9*, *WDR1*
	17	55.52–56.48	1.33	22	*PRKAB1*, *ACAD10*, *CIT*, *HSPB8*
RB (%)	2	131.94–132.93	1.45	20	*PINK1*, *CDA*, *PLA2G2C*
	4	20.30–21.28	2.28	30	*THSD7A*, *TMEM106B*, *ENSBTAG00000067048*
	8	45.67–46.65	2.08	14	*APBA1*, *PTAR1*, *CFAP95*, *SMC5*, *KLF9*
	13	25.27–26.26	1.07	18	*KIAA1217*
	16	71.09–72.03	1.17	24	*RPS6KC1*, *VASH2*, *NSL1*, *BATF3*, *NENF*
	19	7.33–8.33	2.23	21	*ANKFN1*, *NOG*
		36.99–37.95	1.63	15	*COL1A1*, *ITGA3*, *NGFR*, *PHB1*, *SLC35B1*, *SPOP*
	28	8.34–9.22	2.50	21	*TBCE*, *B3GALNT2*, *GNG4*, *LYST*, *NID1*, *GPR137B*, *ERO1B*, *EDARADD*
	29	30.29–31.27	1.35	19	*ETS1*
TPC (%)	4	38.07–39.06	1.12	17	*PCLO*, *CACNA2D1*
	6	105.60–106.57	1.02	22	*SLC2A9*, *WDR1*

^1^ carcass weight (CW), eye muscle area (EMA), backfat thickness (BF), marbling score (MS), tenderloin (TLN), sirloin (SLN), striploin (STLN), chuckroll (CHK), shoulder (SLD), top round (TRD), bottom round (BRD), brisket (BSK), shank (SK), rib (RB), total primal cut yield (TPC); ^2^ *Bos taurus* autosome; ^3^ represents the proportion of genetic variance explained by 1.0 Mb.

## Data Availability

The data presented in this study are available on request from the corresponding author.

## Supplementary Materials

The following supporting information can be downloaded at: https://www.mdpi.com/article/10.3390/ani16010136/s1. Table S1. Explained genetic variance of SNPs within significant windows for CW; Table S2. Explained genetic variance of SNPs within significant windows for EMA; Table S3. Explained genetic variance of SNPs within significant windows for BF; Table S4. Explained genetic variance of SNPs within significant windows for MS; Table S5. Explained genetic variance of SNPs within significant windows for TLN; Table S6. Explained genetic variance of SNPs within significant windows for SLN; Table S7. Explained genetic variance of SNPs within significant windows for STLN; Table S8. Explained genetic variance of SNPs within significant windows for CHK; Table S9. Explained genetic variance of SNPs within significant windows for SLD; Table S10. Explained genetic variance of SNPs within significant windows for TRD; Table S11. Explained genetic variance of SNPs within significant windows for BRD; Table S12. Explained genetic variance of SNPs within significant windows for BSK; Table S13. Explained genetic variance of SNPs within significant windows for SK; Table S14. Explained genetic variance of SNPs within significant windows for RB; Table S15. Explained genetic variance of SNPs within significant windows for TPC; Figure S1. Manhattan plots showing the proportion of genetic variance explained by 1.0 Mb windows for primal cut yields. The red dashed line indicates the significance threshold of 1% genetic variance. gVar (%) represents the percentage of additive genetic variance explained by each genomic window.
