# Supplementary material for: Weighted Single-Step Genome-Wide Association Study Identifies Candidate Genes for Carcass Traits and Primal Cut Yields in Hanwoo Cattle
Source: Animals (Basel). 2026 Jan 3;16(1):136. doi: 10.3390/ani16010136 (PMC12784828; doi:10.3390/ani16010136)
Supplement: Supplementary file 1 [file animals-16-00136-s001.zip › Figure S1. Manhattan plots showing the proportion of genetic variance explained by 1.0 Mb windows for primal cut yields. gVar (%) represents the percentage of additive genetic variance explained by each genomic window.pdf]

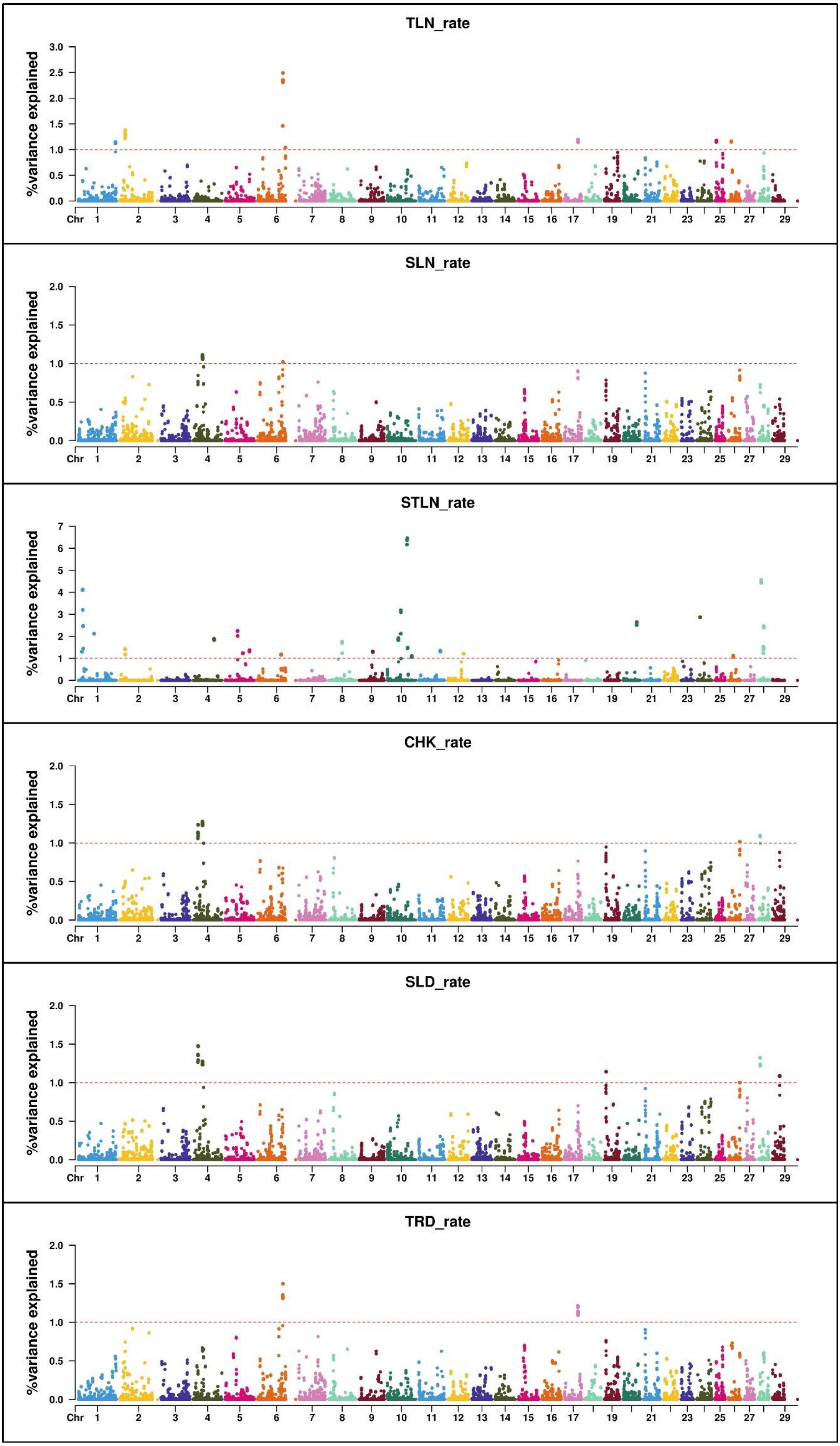
TLN_rate
SLN_rate
STLN_rate
CHK_rate
SLD_rate
TRD_rate
%variance explained
Chr
1 2 3 4 5 6 7 8 9 10 11 12 13 14 15 16 17 19 21 23 25 27 29
0.0 0.5 1.0 1.5 2.0 2.5 3.0
0 1 2 3 4 5 6 7

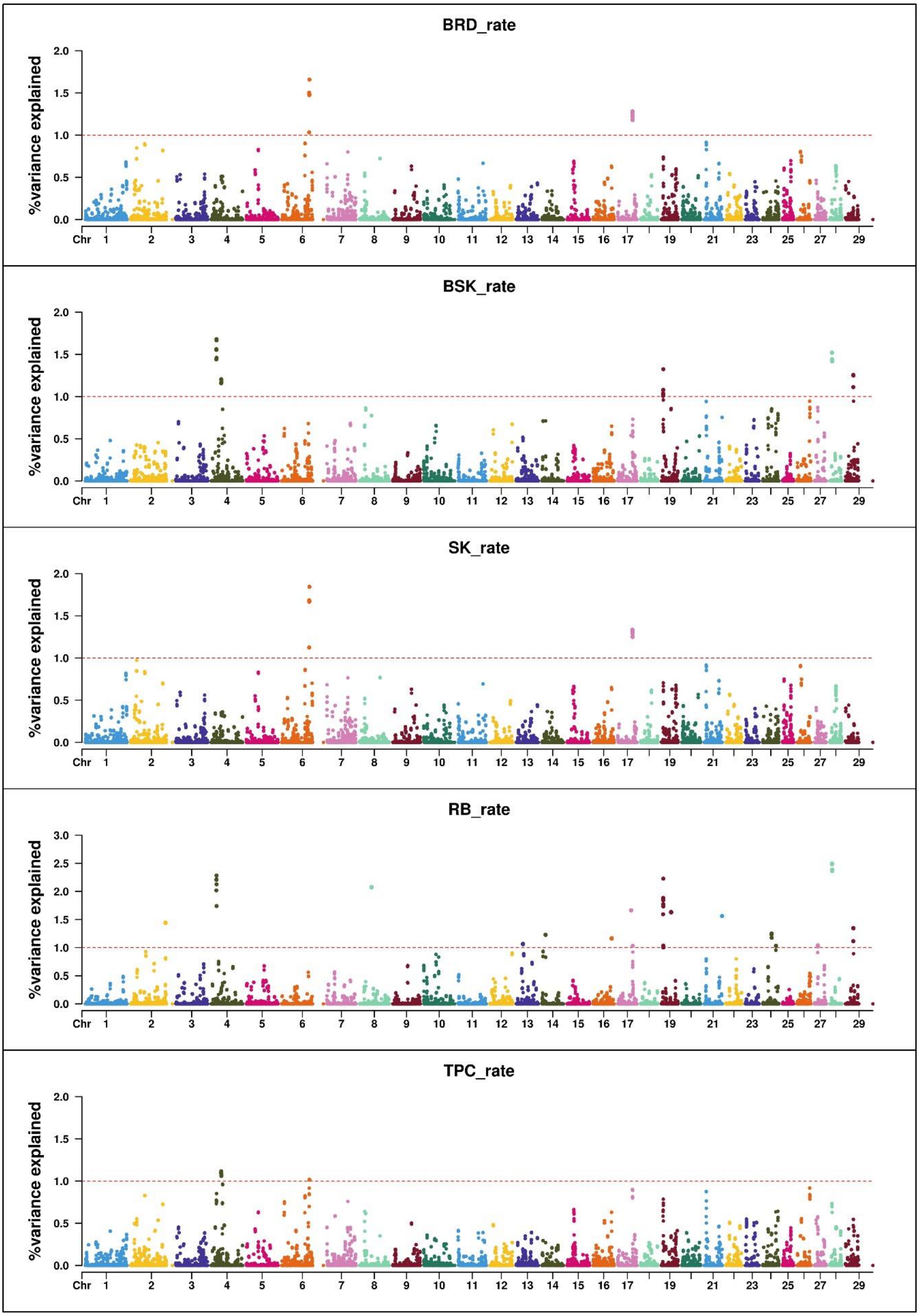


**Fig S1**. Manhattan plots showing the proportion of genetic variance explained by 1.0 Mb windows for primal cut yields. gVar (%) represents the percentage of additive genetic variance explained by each genomic window
